# Safety and immunogenicity of a recombinant interferon-armed RBD dimer vaccine (V-01) for COVID-19 in healthy adults: a randomized, double-blind, placebo-controlled, Phase I trial

**DOI:** 10.1080/22221751.2021.1951126

**Published:** 2021-08-12

**Authors:** Jikai Zhang, Zhongyu Hu, Jianfeng He, Yuyi Liao, Yuan Li, Rongjuan Pei, Xin Fang, Peiyu Zeng, Renfeng Fan, Zhiqiang Ou, Jinglong Deng, Jian Zhou, Wuxiang Guan, Yuanqin Min, Fei Deng, Hua Peng, Zheng Zhang, Chunyan Feng, Baobao Xin

**Affiliations:** a Guangdong Provincial Institute of Biological Products and Materia Medica, Guangzhou, People’s Republic of China; b Guangdong Provincial Center for Disease Control and Prevention, Guangzhou, People’s Republic of China; c National Institutes for Food and Drug Control, Beijing, People’s Republic of China; d Wuhan Institute of Virology, Chinese Academy of Sciences, Wuhan, People’s Republic of China; e Gaozhou Center for Disease Control and Prevention, Maoming, People’s Republic of China; f LivzonBio Inc., Zhuhai, People’s Republic of China; g Key Laboratory of Infection and Immunity, Institute of Biophysics, Chinese Academy of Sciences, Beijing, People’s Republic of China; h Institute for Hepatology, National Clinical Research Center for Infectious Disease, Shenzhen Third People’s Hospital, Shenzhen, People’s Republic of China

**Keywords:** COVID-19, Phase I, clinical trial, recombinant protein vaccine, RBD dimer, safety, immunogenicity, elderly participants

## Abstract

Safe and effective vaccines are still urgently needed to cope with the ongoing COVID-19 pandemic. Recently, we developed a recombinant COVID-19 vaccine (V-01) containing fusion protein (IFN-PADRE-RBD-Fc dimer) as antigen verified to induce protective immunity against SARS-CoV-2 challenge in pre-clinical study, which supported progression to Phase I clinical trials in humans. A Randomized, double-blind, placebo-controlled Phase I clinical trial was initiated at the Guangdong Provincial Center for Disease Control and Prevention (Gaozhou, China) in February 2021. Healthy adults aged between 18 and 59 years and over 60 years were sequentially enrolled and randomly allocated into three subgroups (1:1:1) either to receive the vaccine (10, 25, and 50 μg) or placebo (V-01: Placebo = 4:1) intramuscularly with a 21-day interval by a sentinel and dose escalation design. The data showed a promising safety profile with approximately 25% vaccine-related overall adverse events (AEs) within 30 days and no grade 3 or worse AEs. Besides, V-01 provoked rapid and strong immune responses, elicited substantially high-titre neutralizing antibodies and anti-RBD IgG peaked at day 35 or 49 after first dose, presented with encouraging immunogenicity at low dose (10 μg) subgroup and elderly participants, which showed great promise to be used as all-aged (18 and above) vaccine against COVID-19. Taken together, our preliminary findings indicate that V-01 is safe and well tolerated, capable of inducing rapid and strong immune responses, and warrants further testing in Phase II/III clinical trials.

## Introduction

Coronavirus Disease 2019 (COVID-19) outbreak was declared a pandemic by the World Health Organization (WHO) on 11 March 2020, which has affected millions worldwide [1]. The pandemic has emerged as an enormous threat to public health and caused catastrophic damage to the global economy, triggering severe recessions in many countries. There have been over 171,292,827 confirmed COVID-19 cases worldwide, including 3,687,589 deaths, according to the data revealed by WHO as of 3 June 2021. An accumulated number of 1,581,509,628 vaccine doses have been administered. However, universal access to safe and effective vaccines is currently not available in majority countries and regions, which calls for the capability of rapid scale-up production and applicable storage/handling at cost and condition that allows broad use.

Multiple types of SARS-CoV-2 vaccine candidates have been developed and proceeded into Phase I/IIa clinical trials and even IIb/III clinical trials that tested their efficacy. Immunological effects and safety of these vaccines have been reported [2–4]. The leading vaccine candidates that have been approved for emergency use include inactivated, adenovirus-based, mRNA-based, and recombinant protein vaccines, respectively. All types of SARS-CoV-2 vaccines in the pipeline possess distinct strengths and weaknesses. The traditional inactivated vaccine targets all viral proteins. However, it has been challenging to produce on a large scale in facilities at biosafety level 3 and maintain the intact spike proteins on viral particles to generate high titres of neutralizing antibodies. Monomeric S protein or RBD generally yields low titres of neutralizing antibodies owning to its instability and poor immunogenicity [5,6]. Adenovirus vector-based vaccines encoding viral antigens, such as Ad5-nCoV, stimulate both B cell and T cell responses and are preferably used as a single injection. Pre-existing anti-vector immunity may wreck immune responses and resulting in low neutralization antibody titres and invalid immune boost after repeated vaccination [7–9]. The mRNA-based vaccines are the current leading vaccines due to rapid manufacturability after new outbreaks and induce strong antibody responses and T cell responses, despite the potential safety concerns. The stringent requirements for the manufacturing, storage, transportation, and delivery of mRNA vaccines further limit its broader applicability, especially in developing countries [2,10–12], where COVID-19 vaccines are desperately required. Recombinant protein vaccines based on SARS-CoV-2 S-trimer, RBD-dimer, and RBD-nanoparticles have been developed to generate higher levels of neutralizing antibodies than monomeric proteins [13–16]. In spite of this, an adjuvant is critical for the protein-based vaccine to induce robust immune responses. Compared to commonly used and well-validated adjuvants like alum adjuvant, novel adjuvants, such as Matrix-M and Advax, were conditionally allowed for emergency use in recombinant protein vaccines to stimulate robust anti-viral responses but may be limited by adjuvant availability and the risk of severe side effects [13,17,18].

Current vaccine shortages needed to be solved urgently via developing and promoting new vaccines with properties that are (1) highly effective by achieving high-titre neutralizing antibodies and robust T cell response; (2) safe for all age groups, especially elderly people; (3) capable of producing prolonged protective immunity; and (4) conducive to simplified large-scale production, storage, and distribution. Thus, we previously reported a next-generation fusion-protein vaccine to enhance the immunogenicity without special adjuvants and guaranteed an efficient and safe vaccine [19]. In this vaccine, RBD is armed with an interferon-*α* (IFN*α*) at the N-terminus and dimerized by human IgG1 Fc at the C-terminus (named I-R-F) to target and activate dendritic cells to migrate toward the local draining lymph nodes (LNs), thus enhancing antigen processing and presentation. Low dose I-R-F showed more potent immunogenicity than monomeric RBD in the mouse model, inducing robust antibody titres of balanced IgG1 and IgG2a subtypes and robust CD8+ T cell response, even without additional adjuvant. Further, the addition of a pan HLA-DR-binding epitope (PADRE) in I-R-F (named I-P-R-F or V-01) enhances T cell help-mediated immune response [20]. Further, V-01, intramuscularly injected in the lateral thigh, efficiently provided complete protection in both upper and lower respiratory tracts against a high titre (1 × 10^7^ TCID_50_) SARS-CoV-2 challenge in *Rhesus macaques* [19].

We conducted a randomized, double-blind, placebo-controlled Phase I clinical trial on 24 February 2021, to evaluate the safety and immunogenicity of recombinant COVID-19 fusion protein vaccine (V-01) in healthy subjects. Herein, we report the preliminary assessment of the safety, tolerability, and immunogenicity of V-01 with a 21-day interval two-dose regimen of 10, 25, and 50 μg in healthy adults of both young (18–59 years of age (YOA)) and elder (≥60 YOA) adults in China.

## Methods

### Study design and participants

A randomized, double-blind, placebo-controlled, Phase I trial was initiated on 24 February 2021 at the Guangdong Provincial Center for Disease Control and Prevention (Gaozhou, China). The trial was registered with chictr.org (ChiCTR2100045108). Eligible participants were healthy adults aged over 18 YOA, with a body-mass index of 18–28 kg/m^2^, without a history of travelling in moderate to high-risk areas or a history of contact with confirmed, asymptomatic, or suspected COVID-19 patients. Exclusion criteria were participants with a history of COVID-19 or those who tested positive at screening (by RT–PCR or ELISA); with a history of SARS, autoimmune diseases, severe allergy reactions to vaccines, congenital or acquired angioneurotic oedema; with clinically significant abnormal laboratory test; with an allergy to any ingredients of the vaccine; with confirmed or suspected immunosuppressive or immunodeficiency disorders; who received any blood products in the past 3 months; who received any research medicines or vaccines in the past 6 months; and those being unable to comply with the study schedule. Further details are outlined in the study protocol.

A written informed consent of each participant was obtained by the investigators prior to any study procedure. The trial protocols were approved by the institutional review board of the Guangdong Provincial Center of Disease Control and Prevention and were performed in accordance with the Declaration of Helsinki and Good Clinical Practice. The investigators collected reports of adverse events (AEs) after vaccination and reported to the Data Safety Monitoring Board (DSMB) regularly. The DSMB independently analysed the post vaccination safety data in each dose group, and made suggestions to the sponsor to suspend or terminate the recruiting of participants as and when required during the trials.

### Randomization and masking

Young adult (18–59 YOA, *n* = 90) or elder adult (≥60 YOA, *n* = 90) participants in Phase I trial were randomly assigned (1:1:1) into three subgroups either to receive the vaccine (10, 25, or 50 μg dosage) or placebo at a day 0, 21 schedule. Each subgroup was further divided into V-01 or placebo administration at a ratio of 4:1. A random table was generated by statisticians using SAS statistical software (version 9.4, SAS Institute Inc., USA). Participants were randomly assigned to each group by a randomized block design, with a block of six and rand of five. Investigators assigned random numbers to eligible participants according to the order of the screening sequence. Experimental vaccines or placebos were obtained and administered according to these random numbers. Statisticians were not allowed to disclose the masking code to any personnel in the clinical trials. The vaccine and placebo were identical in appearance. The participants, investigators, and laboratory staffs were all masked to group allocation during the trial.

### Procedures

Our recombinant fusion protein COVID-19 vaccine (V-01) was jointly developed by the Institute of Biophysics, Chinese Academy of Sciences, and Livzon Bio Inc. China. The active component of V-01 is a recombinant fusion protein using RBD dimmer as antigen, whose structure is described in our previous study [19]. The vaccine was manufactured according to good manufacturing practice guidelines. The protein of V-01 was expressed by CHO cells and purified by a couple of steps including chromatography. The formulation is a liquid suspension containing 10, 25, or 50 μg per 0.5 mL/vial, with aluminium hydroxide as the adjuvant. The placebo contains only aluminium hydroxide in solution buffer identical to that of vaccine, without the recombinant fusion protein as antigen. The two doses of vaccine or placebo was administrated intramuscularly in the arm of each participant with a 21-day interval.

To ensure the safety of the participants, the Phase I trial was carried out in a dose-escalating and age-sequential enrolment manner. The study began with the enrolment of five sentinel participants who received the low-dosage (10 μg) vaccine on day 0 and followed by a subsequential 3-day safety observation (including laboratory safety tests). Other subjects of the same dosage were only able to be vaccinated after a safe outcome of the 3-day observation declared by investigators. When the five sentinels of a previous lower dose group were evaluated as safe by the DSMB after a 7-day safety observation, the study was able to proceed into a next higher dose or elder subgroup. During the study, if any safety issues of the vaccine were noted, the recruiting process would be suspended.

In the trial, participants were required for an observation of 30–60 min after each dose of vaccination for treatment-emergent adverse events (TEAE). During the first 7 days after each vaccination, any AE were self-reported by participants daily on the diary cards and verified by investigators. AEs occurring 8-30d/21d (first vaccination) after each vaccination were reported by participants through contact cards. Any serious adverse events (SAEs) occurring in our Phase I trial after the first dose of vaccination were monitored. Solicited local AEs at the injection site within 7d after vaccination included pain, pruritus, redness, swelling, rash, and induration, while solicited systemic AEs within 7d after vaccination included fever, diarrhoea, constipation, dysphagia, anorexia, vomiting, nausea, muscle pain, arthralgia, joint pain, headache, cough, dyspnoea, pruritus (not at the injection site), abnormal skin or mucosa, acute allergic reaction, and fatigue. Laboratory safety tests at the third day after each inoculation, including serum chemistry, haematology, urinalysis, were included in Phase I trial for safety assessment after vaccination. AEs were encoded according to the MedDRA and were graded according to the latest scale issued by the National Medical Products Administration (NMPA) of China.

Serum samples were collected to evaluate RBD-binding capacity and neutralizing activity. Blood samples were taken from participants at the scheduled site visits before vaccination on day 0, 21, 28, 35, and 49 after the first vaccination. Both IgG and IgM antibodies binding capacity to the SARS-CoV-2 RBD protein was determined by the National Institute for Food and Drug control (Beijing China), using ELISA kits (Wantai BioPharm, Beijing, China) according to the manufacturer’s protocol. We also assessed neutralizing activities against live SARS-CoV-2 by microcytopathogenic effect (CPE) assays, which were conducted by the Wuhan Institute of Virology, Chinese Academy of Science. The lower limits of detection for specific RBD-binding IgG antibodies and neutralizing antibodies measurements were 11 and 10, respectively, and those below the detection limit were assigned to 5.5 and 5, respectively.

### Outcomes

The safety outcomes were the frequencies and percentages of AEs within seven days after the vaccination, AEs between the first vaccination to 30 days after the final vaccination, any abnormal changes in laboratory measures at seven days post-vaccination, and SAEs during the whole follow-up period. AEs, including all AEs, AEs related to vaccination, AEs at grade 3 or worse, and AEs leading to the withdrawal of participants, and AEs of special interest in all groups and dose regimens were analysed.

Outcomes for immunogenicity were the seroconversion rate and geometric mean titres (GMTs), geometric mean gold increases (GMIs) of the RBD-binding antibody, SARS-CoV-2 neutralizing antibody. The seroconversion rate was defined as a change from seronegative to seropositive or an at least a 4-fold increase from baseline titres if seropositive at day 0.

### Statistical analysis

Participants with a sample size of 180 in Phase I trial were recruited, which would be sufficient for corresponding safety assessment based on statistical assumptions: a total number of 24 (V-01) in each dose subgroup will be needed to observe a TEAE with an incidence of 8% at least once at 86.5% probability. We did a safety analysis of all participants who received the first dose after enrolment. We did immunogenicity analyses on per-protocol sets, including participants who completed their assigned two-dose regimen and with available antibody results. We presented the frequencies and percentages of participants experiencing each AE post vaccination, and compared safety profiles across the dose groups using Fisher’s exact test. The antibodies against SARS-CoV-2 were presented as GMTs with 95% Clopper–Pearson interval. Correlation analysis of neutralizing antibody and anti-RBD IgG binding antibody was done, and the Pearson correlation coefficient was calculated. We used the *χ*² test or Fisher’s exact test to analyse categorical data, analysis of variance to analyse the log-transformed antibody titres, and Wilcoxon rank-sum test for data that were not normally distributed. All statistical calculations will be processed by SAS 9.4 (SAS Institute Inc., USA) and GraphPad Prism 9.0 (GraphPad Software Inc., San Diego, CA, USA).

## Results

**Figure 1.**
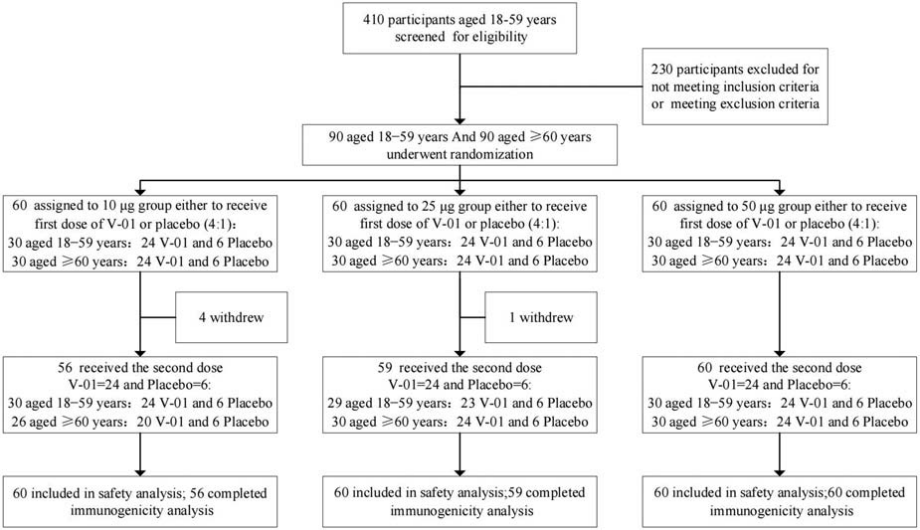
Trial flow diagram.

Between 24 February and 29 March 2021, 410 individuals were screened, and 180 eligible participants were enrolled in the Phase I trial (Figure 1). Participants were sequentially enrolled and randomly allocated into three subgroups (1:1:1) either to receive the vaccine (10, 25, and 50 μg) or placebo with a ratio of 4:1, which was intramuscularly administered with a 21-day interval. Four participants in the ≥60 YOA group (10 μg V-01) and one participant in the 18–59 YOA group (25 μg V-01) withdrew before receiving the second dose. The participants’ characteristics (Table 1) were comparable across the treatment groups both in younger and elder adults with mean age ranging from 40.0 to 44.5 years and from 64.8 to 65.8 years, and mean BMI ranging from 22.9 to 24.5 kg/m^2^ and from 22.6 to 23.2 kg/m^2^, respectively. However, the participants showed no ethnic diversity, with 100% of participants being Han Chinese and presented an unbalanced gender distribution across the treatment groups, particularly the elder adult participants were predominantly males.

**Table 1.** Baseline characteristics of the participants classified by age.

	Younger adults (18–59)				Elder adults (≥60)			
	10 μg (*n* = 24)	25 μg (*n* = 24)	50 μg (*n* = 24)	Placebo (*n* = 18)	10 μg (*n* =24)	25 μg (*n* = 24)	50 μg (*n* = 24)	Placebo (*n* = 18)
Age (y)								
Mean (SD)	40.0 (9.2)	41.6 (12.1)	44.5 (10.6)	40.7 (11.3)	65.8 (4.4)	65.0 (2.9)	65.9 (4.0)	64.8 (4.4)
Ethnicity (%)								
Han Chinese	24 (100)	24 (100)	24 (100)	18 (100)	24 (100)	24 (100)	24 (100)	18 (100)
Sex (%)								
Male	6 (25.00)	11 (45.83)	14 (58.33)	10 (55.56)	16 (66.67)	20 (83.33)	19 (79.17)	18 (100.00)
Female	18 (75.00)	13 (54.17)	10 (41.67)	8 (44.44)	8 (33.33)	4 (16.67)	5 (20.83)	0 (0.00)
BMI (kg/m^2^)								
Mean (SD)	23.2 (3.0)	22.9 (2.6)	24.5 (2.1)	23.1 (2.5)	22.9 (2.4)	22.6 (3.1)	23.1 (2.3)	23.2 (2.7)

Note: Data are presented as mean (SD) or n (%). BMI = body-mass index.

Our preliminary safety data exhibited a promising safety profile with approximately 25% (46 out of 180) vaccination-related AEs and no grade 3 or worse AEs. We observed no significant differences of overall AEs within 30 d across prophylaxis groups and placebo groups in our age-stratified safety data, with a percentage of 66.67%, 70.83%, 79.17% vs 72.22%, and 54.17%, 54.17%, 62.50% vs 38.89%, respectively (Table 2). All the AEs were mild or moderate in severity within 30 days after inoculation. The most common solicited local AEs were pain and pruritus at the injection site. The most common solicited systemic AEs were fever, anorexia, muscle pain, headache. No AEs of special interest were observed, and no AEs leading to the withdrawal of participants were reported.

**Table 2.** AEs in Phase 1 trial.

	Younger adults (18–59)					Elder adults (≥60)				
AEs	10 μg (*n* = 24)	25 μg (*n* = 24)	50 μg (*n* = 24)	Placebo (*n* = 18)	*P* value	10 μg (*n* = 24)	25 μg (*n* =24)	50 μg (*n* = 24)	Placebo (*n* = 18)	*P* value
*Overall AEs within 30 d*										
Any^a^	16 (66.67)	17 (70.83)	19 (79.17)	13 (72.22)	0.8153	13 (54.17)	13 (54.17)	15 (62.50)	7 (38.89)	0.5328
Vaccination-related^b^	7 (29.17)	9 (37.5)	10 (41.67)	5 (27.78)	0.7787	2 (8.33)	4 (16.67)	7 (29.17)	2 (11.11)	0.2760
Grade ≥3	0	0	0	0	1.0000	0	0	0	0	1.0000
*Solicited local adverse reactions*										
Pain	2 (8.33)	2 (8.33)	4 (16.67)	4 (22.22)	0.5182	0	0	1 (4.17)	0	1.0000
Induration	0	0	0	0	1.0000	0	0	0	0	1.0000
Swelling	0	0	0	0	1.0000	0	0	0	0	1.0000
Rash	0	0	0	0	1.0000	0	0	0	0	1.0000
Redness	0	0	0	0	1.0000	0	0	0	0	1.0000
Pruritus	1 (4.17)	0	0	0	1.0000	0	0	0	0	1.0000
*Solicited systemic adverse reactions*										
Fever	2 (8.33)	1 (4.17)	1 (4.17)	0	0.9026	0	0	0	0	1.0000
Diarrhoea	0	0	0	0	1.0000	0	0	0	0	1.0000
Constipation	0	0	0	0	1.0000	0	0	0	0	1.0000
Dysphagia	0	0	0	0	1.0000	0	0	0	0	1.0000
Anorexia	2 (8.33)	1 (4.17)	1 (4.17)	0	0.9026	0	0	0	0	1.0000
Vomiting	1 (4.17)	0	0	0	1.0000	0	0	0	0	1.0000
Nausea	2 (8.33)	0	0	1 (5.56)	0.2449	0	0	0	0	1.0000
Muscle pain	0	0	2 (8.33)	1 (5.56)	0.2793	0	0	0	0	1.0000
Arthralgia	0	0	0	1 (5.56)	0.2000	0	0	0	0	1.0000
Headache	1 (4.17)	0	0	1 (5.56)	0.5685	0	0	2 (8.33)	0	0.2449
Cough	0	0	1 (4.17)	1 (5.56)	0.5685	0	1 (4.17)	0	0	1.0000
Dyspnoea	0	0	0	0	1.0000	0	0	0	0	1.0000
Pruritus	0	0	0	1 (5.56)	0.2000	0	0	0	0	1.0000
Abnormal skin or mucosa	0	0	0	0	1.0000	0	0	0	0	1.0000
Acute allergic reaction	0	0	0	0	1.0000	0	0	0	0	1.0000
Fatigue	2 (8.33)	2 (8.33)	3 (12.50)	3 (16.67)	0.8832	0	1 (4.17)	3 (12.50)	0	0.2431
*Unsolicited adverse reactions*										
Any	15 (62.50)	15 (62.50)	147 (70.83)	11 (61.11)	0.9102	12 (50.00)	12 (50.00)	15 (62.50)	7 (38.89)	0.5165
Vaccination-related	5 (20.83)	5 (20.83)	4 (16.67)	1 (5.56)	0.5269	2 (8.33)	3 (12.50)	6 (25.00)	2 (11.11)	0.4809

Notes: Data are presented as *n* (%). *P* values are calculated with Fisher’s exact test.

^a^ *Any* means the total number of AEs within 30days regardless of the causal relationship between the adverse events and vaccination.

^b^ *Vaccination-related* means adverse events within 30days was correlated with vaccination judged by the investigators.

**Figure 2.**
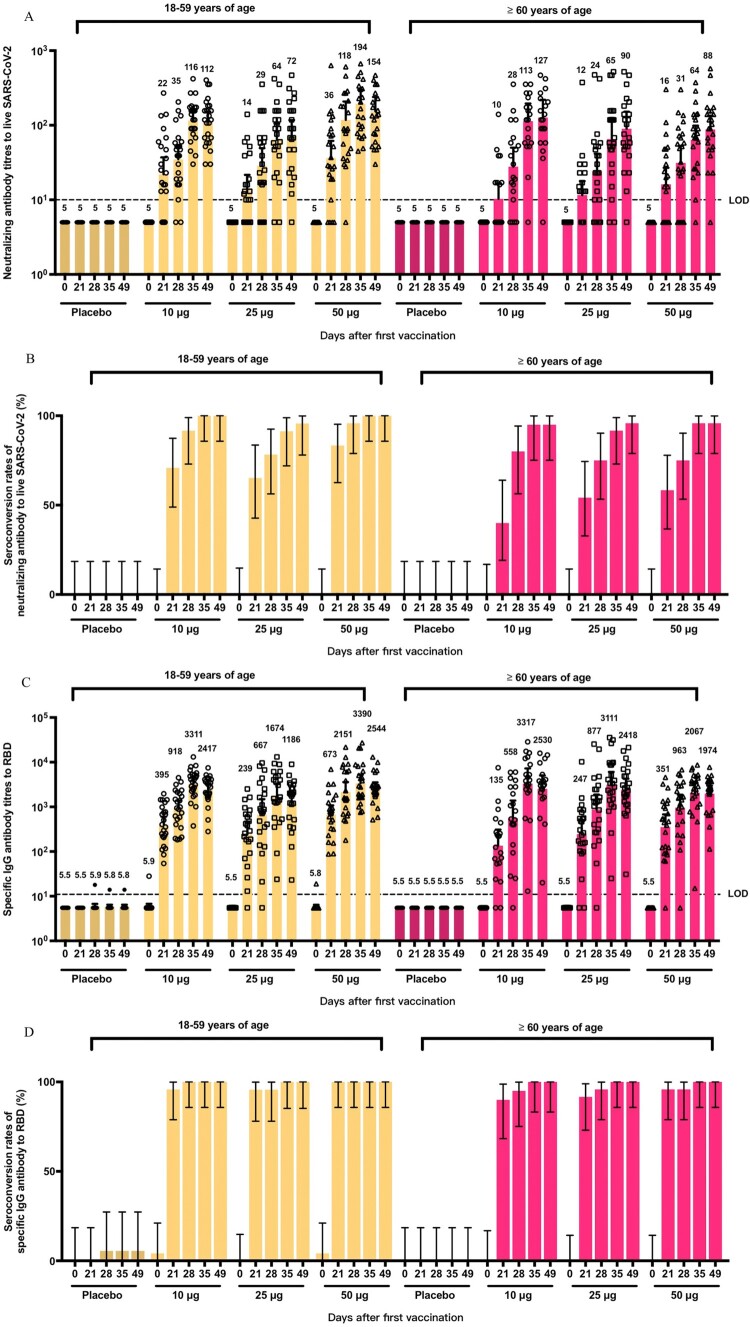
Humoral immune responses in phase I trials. GMTs (A) and seroconversion rates (B) of neutralizing antibodies at different timepoints after first vaccination in phase 1. GMTs (C) and seroconversion rates (D) of RBD-binding antibodies at different timepoints after first vaccination in phase 1. Error bars represent 95% CIs of geomeans. The horizontal dashed lines in panels A and C indicate the limit of detection. RBD: receptor-binding domain.

Neutralization antibody titres to live SARS-CoV-2 as well as RBD-binding antibody titres were assessed at day 0 (baseline titres, immediately before the first dose), day 21 (immediately before the second dose), day 28, day 35, and day 49. All the participants were seronegative at baseline, had a modest vaccine-induced immune response at day 21, further increased at day 28, peaked at day 35 or day 49 (Figure 2). Neutralizing antibodies with a GMT of 112.2 (95%CI: 82.27–153), 71.6 (95%CI: 43.58–117.5), 154.2 (95%CI: 99.46–239.1) from younger adults and 126.9 (95%CI: 73.62–218.8), 89.9 (95%CI: 53.27–151.6), 87.7 (95%CI: 57.2–134.6) from elder adults were noted in the 10, 25, and 50 μg V-01 group, respectively. A robust immune response has been evaluated in the low-dose group. We also observed a substantially higher GMT at day 49, which was a 2.5- to –5.4-fold increase as compared with that at day 21, indicating a boosting humoral immune response after a second dose. Consequently, seroconversion rates of neutralization antibody were mostly below 70% at day 21 and were above 95% in all vaccinated groups at day 49.

A similar pattern was identified in the RBD-binding antibody as compared with live SARS-CoV-2 neutralization antibody titres (Figure 2). The seroconversion rates of RBD-binding antibody were encouragingly above 90% at day 21 and were predominantly 100% in vaccinated groups at day 35 and day 49 except for younger adults at 25 μg. Above all, V-01 demonstrated satisfactory RBD-binding capacity in an elder adult group with a GMT of 3317 (95%CI: 1518–7249), 3111 (95%CI: 1596–6062), 2067 (95%CI: 1190–3590) versus 3311 (95%CI: 2391–4586), 1674 (95%CI: 863–3246), 3390 (95%CI: 2196–5234) in younger adults at day 35, which shown promising potential to be an all-aged vaccine candidate against COVID-19. Meanwhile, the specific IgM antibody binding to RBD was also measured. The results showed that approximately 30% to 80% participants in V-01 groups elicited IgM responses against V-01 administration (Figures S1 and S2).

**Figure 3.**
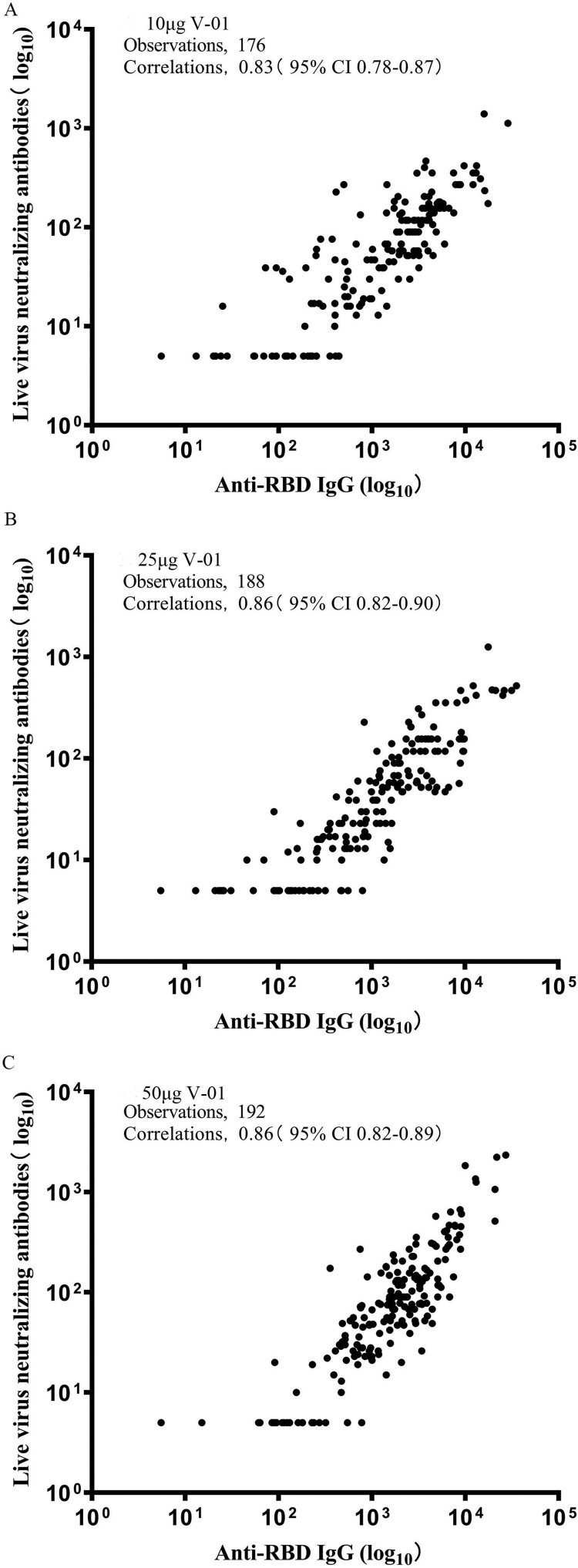
Correlation of live virus neutralizing antibodies and anti-RBD IgG binding antibody response. Scatter plots of log_10_ transformed live SARS-COV-2 neutralizing antibody responses and anti-RBD IgG binding antibody responses at day 21 (immediately before the second dose), day 28 (1 week after the second vaccination), day 35 and day 49 in the 10 (A), 25 (B), and 50 μg (C) V-01 group.

A strong correlation was observed between live virus-neutralizing antibody titres and anti-RBD IgG binding antibody titres, and the correlation coefficient was 0.83 (95%CI: 0.78–0.87), 0.86 (95%CI: 0.82–0.90), 0.86 (95%CI: 0.82–0.89) for each dose group, respectively (Figure 3).

## Discussion

The preliminary safety analyses indicate that in healthy adult participants aged over 18 years, with the day 0 and day 21 two-dose regimens of 10, 25, and 50 μg V-01 were considered safe and well tolerated. Recombinant protein vaccines have relatively safe profiles, because this kind of vaccines does not require lipidated PEG to form nanoparticles for delivery, or the adenovirus vectors for viral gene packaging, given that a number of AEs and SAEs present a strong correlation with lipid-PEG formulation and adenovirus vectors [21,22]. The good safety profile of V-01 in Phase I has demonstrated the combination of the recombinant fusion protein and alum adjuvant was successful and reasonable. All the different doses of V-01, adjuvanted with aluminium hydroxide, did not show any grade 3 or worse AEs during Phase I trial. The vaccine-related AEs slightly responded in a reasonable dose-dependent manner in both younger and elder adult groups, which was in agreement with the strategic composition and the molecular design of V-01.

In this work, we have demonstrated that this novel-designed recombinant protein COVID-19 vaccine V-01 induced rapid and strong immune responses during the Phase I clinical trial. Insufficient immune response is generally a disadvantage of protein-based vaccines if the protein antigen has a small size and provokes low immunogenicity, subject to impact from antigenic drift than full-length spike protein. To solve this problem, the SARS-CoV-2 recombinant protein vaccines are using multi-valent RBD of spike protein as the antigen to effectively simulate the immune system. We have previously reported a unique armed RBD dimer protein vaccine (V-01), which elicited high antibody titre in the pre-clinical study [19]. All the three groups that were administered with varying dosage achieved high immunogenicity presented with substantially high titres of neutralizing antibodies and RBD-binding antibodies. The fact that the lowest dose (10 μg) also elicited immunogenicity, indicates the robustness of the immune-response properties of V-01. In comparison with the previous reports from other recombinant protein vaccines, the dose range of V-01 (10–50μg) is reasonable and similar to that of existing leading recombinant vaccines [23–25]. However, it is yet not known why the titre of neutralizing antibody and RBD binding IgG behaved in a dose-independent manner, with relatively higher titre at 10 and 50 μg dosage, while lower at 25 μg dosage in the group of 18–59 YOA. A detailed mechanistic understanding will vastly aid the development of recombinant vaccines. V-01 elicited a fast immune response in this trial with an RBD binding IgG titre of 239–673 on day 21 after a single injection and 1674–3390 on day 35, which peaked at 14-day after a second inoculation. Rapid immune response is consistent with our pre-clinical study [19] with high RBD-specific IgG endpoint titre (>10^5^) generated by either high dose (50 μg) or low dose (10 μg) vaccination on day 21 in *Rhesus macaques*, which will play an important and indispensable role in providing protective immunity for a current worldwide pandemic, especially in some developing countries and the outbreak regions. One week or several days, early effective protection of these “hot zones” could significantly control the spread tendency. Neutralizing antibodies to SARS-CoV-2 are typically of more interest while determining vaccine-induced protection, although the correlations between neutralizing titres and protection efficacy are still hard to predict. Here, we noted that neutralizing antibodies with a geometric mean titre of 114.8 (95%CI: 87.79-150.1), 113.4 (95%CI: 65.89–195.3) at the 10 μg V-01 group (14-day after the second dose) in younger and elder adults, respectively. The values were within the higher range of neutralizing antibody titres in the reported COVID-19 vaccines.

The robust immunogenicity of V-01 is based on its molecular design, which has been discussed in our pre-clinical studies [19]. Our findings further demonstrated that interferon-armed RBD dimer truly stand out from the whole spike protein as the antigen to be processed and presented with IFN*α*-mediated, dendritic cells and follicular T-helper lymphocytes (Tfh) involved immunological priming pathway. PADRE sequence was intended for the stimulation of Th cell, and the Fc fragment for the long-lasting effect. We used a common, well-established alum adjuvant known to potentiate the immune response by promoting the uptake of antigens via antigen-presenting cells, with a good track record of safety and low cost for broad access.

Moreover, V-01 induced substantial humoral response in the elderly participants (≥60 years), which is rarely recruited in the COVID-19 vaccine Phase I clinical trials. The anti-RBD IgG titre of V-01 in the elder group was 135–351 on day 21 and peaked on day 35 with a value of 2067-3317. The neutralizing antibody titre to live SARS-CoV-2 ranged 64–113 on day 35, and 88–127 on day 49. The vaccine-related AEs displayed lower frequency in the ≥60 years-old group than those in the 18–59 years-old group, indicating that reactogenicity in V-01 could be absent or mild to elder participants. Therefore, the encouraging safety and immunogenicity results suggest that V-01 could be a valuable prophylactic vaccine candidate for general elderly people, who are at higher risk and larger budget for developing severe or critical cases during the COVID-19 pandemic.

This trial also has several limitations:(1) Since diverse assays for measuring the neutralizing antibody are used in different trials, it is difficult to evaluate the different vaccines in a standard procedure. Besides, the control of human convalescent serum from COVID-19 patients is lacked in this study, which is incapable of clarifying the correlation between neutralizing antibody GMT and protection. (2) This trial only recruited adults over 18 years, which is short of the immunogenicity and safety data in the younger aged group. (3) The immune persistence has not been observed in this trial, which could be arranged in the trial II/III studies.

In summary, remarkable safety profile with rapid and strong immunogenicity of V-01 strongly warrants further testing in Phase II/III clinical studies. Owing to the limited sample size in Phase I, an appropriate dose regimen for Phase III efficacy study will be determined taking into consideration both immunogenicity and safety results at Phase II. Moreover, V-01 with its unique molecular design, displayed well tolerability and favourable immunogenicity profile in the elderly people, which may be a promising candidate that is suitable for all people above 18 years of age.

## Supplementary Material

Supplementary_information_0627-Revision_finalized_2.docx
