# Supplementary material for: Safety and immunogenicity of a recombinant interferon-armed RBD dimer vaccine (V-01) for COVID-19 in healthy adults: a randomized, double-blind, placebo-controlled, Phase I trial
Source: Emerg Microbes Infect. 2021 Aug 12;10(1):1589–97. doi: 10.1080/22221751.2021.1951126 (PMC8366678; doi:10.1080/22221751.2021.1951126)
Supplement: Supplementary_information_0627-Revision_finalized_2.docx [file TEMI_A_1951126_SM1475.docx]

Table S1. Specific IgG and IgM antibody responses to RBD, neutralizing antibodies to live virus at baseline

|  | **18～59 years of age** | | | | **≥60 years of age** | | | |
| --- | --- | --- | --- | --- | --- | --- | --- | --- |
|  | **10 μg**  **(N=24)** | **25 μg**  **(N=23)** | **50 μg**  **(N=24)** | **Placebo**  **(N=18)** | **10 μg**  **(N=20)** | **25 μg**  **(N=24)** | **50 μg**  **(N=24)** | **Placebo**  **(N=18)** |
| **Neutralizing antibody to live SARS-CoV-2** | | | | | | | | |
| **Seroconversion** | 0  (0.00%, 0.00-14.25) | 0  (0.00%, 0.00-14.82) | 0  (0.00%, 0.00-14.25) | 0  (0.00%, 0.00-18.53) | 0  (0.00%, 0.00-16.84) | 0  (0.00%, 0.00-14.25) | 0  (0.00%, 0.00-14.25) | 0  (0.00%, 0.00-18.53) |
| **GMT** | 5.0 (5.0-5.0) | 5.0 (5.0-5.0) | 5.0 (5.0-5.0) | 5.0 (5.0-5.0) | 5.0 (5.0-5.0) | 5.0 (5.0-5.0) | 5.0 (5.0-5.0) | 5.0 (5.0-5.0) |
| **RBD-IgG** | | | | | | | | |
| **Seroconversion** | 1  (4.17%, 0.11-21.12) | 0  (0.00%, 0.00-14.82) | 1  (4.17%, 0.11-21.12) | 0  (0.00%, 0.00-18.53) | 0  (0.00%, 0.00-16.84) | 0  (0.00%, 0.00-14.25) | 0  (0.00%, 0.00-14.25) | 0  (0.00%, 0.00-18.53) |
| **GMT** | 5.89 (5.12, 6.77) | 5.50 (5.50, 5.50) | 5.79 (5.20, 6.44) | 5.50 (5.50, 5.50) | 5.50 (5.50, 5.50) | 5.50 (5.50, 5.50) | 5.50 (5.50, 5.50) | 5.50 (5.50, 5.50) |
| **RBD-IgM** | | | | | | | | |
| **Seropositive** | 0  (0.00%, 0.00-14.25) | 1  (4.17%, 0.11-21.12) | 0  (0.00%, 0.00-14.25) | 0  (0.00%,0.00-18.53 ) | 0  (0.00%, 0.00-16.84) | 0  (0.00%, 0.00-14.25) | 0  (0.00%, 0.00-14.25) | 0  (0.00%, 0.00-18.53) |
| **GMT** | 5.50 (5.50-5.50) | 6.93 (4.30-11.19) | 5.50 (5.50-5.50) | 5.50 (5.50-5.50) | 5.50 (5.50-5.50) | 5.50 (5.50-5.50) | 5.50 (5.50-5.50) | 5.50 (5.50-5.50) |

Data are presented as GMT (95% CI), number of participants (%, 95% CI) for seroconversion.

Table S2. GMT and seroconversion of specific IgG antibody responses to RBD at various time points in Phase I Trail

|  | **18～59 years of age** | | | | **≥60 years of age** | | | |
| --- | --- | --- | --- | --- | --- | --- | --- | --- |
|  | **10 μg**  **(N=24)** | **25 μg**  **(N=23)** | **50 μg**  **(N=24)** | **Placebo**  **(N=18)** | **10 μg**  **(N=20)** | **25 μg**  **(N=24)** | **50 μg**  **(N=24)** | **Placebo**  **(N=18)** |
| **Day 21** | | | | | | | | |
| **Seroconversion** | 23  (95.83%, 78.88-99.89) | 22  (91.67%, 73.00-98.97) | 24  (100.00%, 85.75-100.00) | 0  (0.00%, 0.00-18.53) | 18  (90.00%, 68.30-98.77) | 22  (91.67%, 73.00-98.97) | 23  (95.83%, 78.88-99.89) | 0  (0.00%, 0.00-18.53) |
| **GMT** | 394.82 (257.61-605.12) | 239.47 (122.30-468.91) | 673.46 (420.06-1079.74) | 5.50 (5.50-5.50) | 135.44 (59.16-310.11) | 247.47 (121.98-502.06) | 351.21 (181.59-679.27) | 5.50  (5.50, 5.50) |
| **Day 28** | | | | | | | | |
| **Seroconversion** | 24  (100.00%, 85.75-100.00) | 22  (91.67%, 73.00-98.97) | 24  (100.00%, 85.75-100.00) | 1  (5.56%, 0.14-27.29) | 19  (95.00%, 75.13-99.87) | 23  (95.83%, 78.88-99.89) | 23  (95.83%, 78.88-99.89) | 0  (0.00%, 0.00-18.53) |
| **GMT** | 918.18 (607.54-1387.64) | 666.59 (300.18-1480.27) | 2150.68 (1287.16-3593.50) | 5.87 (5.11-6.75) | 557.80 (223.60-1391.50) | 876.54 (406.97-1887.88) | 963.31 (494.29-1877.40) | 5.50  (5.50, 5.50) |
| **Day 35** | | | | | | | | |
| **Seroconversion** | 24  (100.00%, 85.75-100.00) | 23  (95.83%, 78.88-99.89) | 24  (100.00%, 85.75-100.00) | 1  (5.56%, 0.14-27.29) | 20  (100.00%, 83.16-100.00) | 24  (100.00%, 85.75-100.00) | 24  (100.00%, 85.75-100.00) | 0  (0.00%, 0.00-18.53) |
| **GMT** | 3311.19 (2391.00-4585.52) | 1673.61 (863.01-3245.59) | 3390.37 (2196.15-5234.00) | 5.79 (5.19-6.46) | 3316.86 (1517.75-7248.59) | 3110.79 (1596.42-6061.72) | 2067.25 (1190.27-3590.39) | 5.50  (5.50, 5.50) |
| **Day 49** | | | | | | | | |
| **Seroconversion** | 24  (100.00%, 85.75-100.00) | 23  (95.83%, 78.88-99.89) | 24  (100.00%, 85.75-100.00) | 1  (5.56%, 0.14-27.29) | 20  (100.00%, 83.16-100.00) | 24  (100.00%, 85.75-100.00) | 24  (100.00%, 85.75-100.00) | 0  (0.00%, 0.00-18.53) |
| **GMT** | 2417.39 (1757.79-3324.51) | 1186.08 (661.76-2125.81) | 2544.49 (1830.96-3536.07) | 5.79 (5.19-6.46) | 2529.58 (1266.73-5051.39) | 2417.85 (1370.75-4264.82) | 1974.32 (1310.28-2974.89) | 5.50 (5.50, 5.50) |

Data are presented as GMT (95% CI), number of participants (%, 95% CI) for seroconversion.

Table S3. GMT and seroconversion of neutralizing antibodies to live virus at various time points in Phase I Trail

|  | **18～59 years of age** | | | | **≥60 years of age** | | | |
| --- | --- | --- | --- | --- | --- | --- | --- | --- |
|  | **10 μg**  **(N=24)** | **25 μg**  **(N=23)** | **50 μg**  **(N=24)** | **Placebo**  **(N=18)** | **10 μg**  **(N=20)** | **25 μg**  **(N=24)** | **50 μg**  **(N=24)** | **Placebo**  **(N=18)** |
| **Day 21** | | | | | | | | |
| **Seroconversion** | 17  (70.83%, 48.91-87.38) | 15  (65.22%, 42.73-83.62) | 20  (83.33%, 62.62-95.26) | 0  (0.00%, 0.00-18.53) | 8  (40.00%, 19.12-63.95) | 13  (54.17%, 32.82-74.45) | 14  (58.33%, 36.64-77.89) | 0  (0.00%, 0.00-18.53) |
| **GMT** | 22.0 (13.0-37.0) | 13.9 (8.9-21.6) | 36.2 (21.2-61.8) | 5.0 (5.0-5.0) | 10.3 (6.4-16.7) | 11.6 (7.5-17.8) | 16.2 (9.7-26.9) | 5.0 (5.0-5.0) |
| **Day 28** | | | | | | | | |
| **Seroconversion** | 22  (91.67%, 73.00-98.97) | 18  (78.26%, 56.30-92.54) | 23  (95.83%, 78.88-99.89) | 0  (0.00%, 0.00-18.53) | 16  (80.00%, 56.34-94.27) | 18  (75.00%, 53.29-90.23) | 18  (75.00%, 53.29-90.23) | 0  (0.00%, 0.00-18.53) |
| **GMT** | 34.9 (23.1-52.8) | 28.6 (16.6-49.2) | 117.6 (66.5-207.9) | 5.0 (5.0-5.0) | 27.8 (15.4-50.0) | 24.0 (13.8-41.5) | 31.3 (18.4-53.2) | 5.0 (5.0-5.0) |
| **Day 35** | | | | | | | | |
| **Seroconversion** | 24  (100.00%, 85.75-100.00) | 21  (91.30%, 71.96-98.93) | 24  (100.00%, 85.75-100.00) | 0  (0.00%, 0.00-18.53) | 19  (95.00%, 75.13-99.87) | 22  (91.67%, 73.00-98.97) | 23  (95.83%, 78.88-99.89) | 0  (0.00%, 0.00-18.53) |
| **GMT** | 116.1 (88.9-151.7) | 64.0 (38.9-105.1) | 194.2 (126.7-297.7) | 5.0 (5.0-5.0) | 113.4 (65.9-195.3) | 65.2 (38.1-111.4) | 63.8 (41.3-98.6) | 5.0 (5.0-5.0) |
| **Day 49** | | | | | | | | |
| **Seroconversion** | 24  (100.00%, 85.75-100.00) | 22  (95.65%, 78.05-99.89) | 24  (100.00%, 85.75-100.00) | 0  (0.00%, 0.00-18.53) | 19  (95.00%, 75.13-99.87) | 23  (95.83%, 78.88-99.89) | 23  (95.83%, 78.88-99.89) | 0  (0.00%, 0.00-18.53) |
| **GMT** | 112.2 (82.3-153.0) | 71.6 (43.6-117.5) | 154.2 (99.5-239.1) | 5.0 (5.0-5.0) | 126.9 (73.6-218.8) | 89.9 (53.3-151.6) | 87.7 (57.2-134.6) | 5.0 (5.0-5.0) |

Data are presented as GMT (95% CI), number of participants (%, 95% CI) for seroconversion.

Table S4. GMT and seroconversion of specific IgM antibody responses to RBD at various timepoints in Phase I Trail

|  | **18～59 years of age** | | | | **≥60 years of age** | | | | |
| --- | --- | --- | --- | --- | --- | --- | --- | --- | --- |
|  | **10 μg**  **(N=24)** | **25 μg**  **(N=23)** | **50 μg**  **(N=24)** | **Placebo**  **(N=18)** | **10 μg**  **(N=20)** | **25 μg**  **(N=24)** | **50 μg**  **(N=24)** | **Placebo**  **(N=18)** |  |
| **Day 21** | | | | | | | | | |
| **Seropositive** | 13  (54.17%, 32.82-74.45) | 11  (47.83%, 26.82-69.41) | 19  (79.17%, 57.85-92.87) | 0  (0.00%, 0.00-18.53) | 6  (30.00%, 11.89-54.28) | 13  (54.17%, 32.82-74.45) | 13  (54.17%, 32.82-74.45) | 0  (0.00%, 0.00-18.53) |  |
| **GMT** | 28.82  (13.02-63.78) | 17.15 (9.23-31.89) | 70.14 (29.05-169.38) | 5.50 (5.50-5.50) | 9.63  (5.80-15.99) | 23.27  (10.71-50.60) | 25.54 (11.51-56.71) | 5.50 (5.50-5.50) |  |
| **Day 28** | | | | | | | | | |
| **Seropositive** | 14  (58.33%, 36.64-77.89) | 11  (47.83%, 26.82-69.41) | 18  (75.00%, 53.29-90.23) | 0  (0.00%, 0.00-18.53) | 8  (40.00%, 19.12-63.95) | 12  (50.00%, 29.12-70.88) | 13  (54.17%, 32.82-74.45) | 0  (0.00%, 0.00-18.53) |  |
| **GMT** | 22.10  (11.00-44.41) | 15.29 (8.64-27.06) | 47.79 (20.94-109.08) | 5.50 (5.50-5.50) | 11.60  (6.47-20.80) | 21.93  (9.86-48.79) | 23.41 (11.09-49.42) | 5.50 (5.50-5.50) |  |
| **Day 35** | | | | | | | | | |
| **Seropositive** | 14  (58.33%, 36.64-77.89) | 10  (43.48%, 23.19-65.51) | 17  (70.83%, 48.91-87.38) | 0  (0.00%, 0.00-18.53) | 12  (60.00%, 36.05-80.88) | 13  (54.17%, 32.82-74.45) | 12  (50.00%, 29.12-70.88) | 0  (0.00%, 0.00-18.53) |  |
| **GMT** | 21.26  (11.29-40.04) | 12.01 (7.83-18.41) | 37.12 (17.06-80.78) | 5.50 (5.50-5.50) | 19.79  (9.82-39.89) | 22.73  (10.29-50.22) | 17.29  (9.39-31.86) | 5.50 (5.50-5.50) |  |
| **Day 49** | | | | | | | | | |
| **Seropositive** | 12  (50.00%, 29.12-70.88) | 8  (34.78%, 16.38-57.27) | 15  (62.50%, 40.59-81.20) | 0  (0.00%, 0.00-18.53) | 7  (35.00%, 15.39-59.22) | 9  (37.50%, 18.80-59.41) | 10  (41.67%, 22.11-63.36) | 0  (0.00%, 0.00-18.53) |  |
| **GMT** | 17.32  (8.70-34.49) | 8.58  (6.51-11.31) | 21.87 (10.91-43.84) | 5.50 (5.50-5.50) | 11.78  (6.60-21.03) | 17.13  (8.26-35.52) | 12.95  (7.81-21.48) | 5.50 (5.50-5.50) |  |

Data are presented as GMT (95% CI), number of participants (%, 95% CI) for seropositive.

Table S5 Adverse events/reactions after each dose

|  | Younger adults (18~59) | | | | | Elder adults (≥60) | | | | |
| --- | --- | --- | --- | --- | --- | --- | --- | --- | --- | --- |
| Adverse events | 10μg | 25μg | 50μg | Placebo | P value | 10μg | 25μg | 50μg | Placebo | P value |
| **Overall adverse events within 30 days** | | | | | | | | |  |  |
| 1st dose | 12/24(50.00) | 13/24(54.17) | 16/24(66.67) | 10/18(55.56) | 0.7019 | 11/24(45.83) | 11/24(45.83) | 12/24(50.00) | 7/18(38.89) | 0.9187 |
| 2nd dose | 10/24(41.67) | 7/24(30.43) | 8/24(33.33) | 7/18(38.89) | 0.8641 | 4/20(20.00) | 3/24(12.50) | 7/24(29.17) | 2/18(11.11) | 0.4471 |
| **Solicited adverse reactions within 7 days** | | | | | | | | | | |
| 1st dose | 2/24(8.33) | 3/24(12.50) | 5/24(20.83) | 5/18(27.78) | 0.3233 | 0 | 1/24(4.17) | 3/24(12.50) | 0 | 0.2008 |
| 2nd dose | 2/24(8.33) | 1/24(4.35) | 3/24(12.50) | 1/18(5.56) | 0.8363 | 0 | 1/24(4.17) | 0 | 0 | 1.0000 |
| **Solicited systemic adverse reactions** | | | | | | | | | | |
| 1st dose | 2/24(8.33) | 2/24(8.33) | 2/24(8.33) | 4/18(22.22) | 0.6073 | 0 | 1/24(4.17) | 3/24(12.50) | 0 | 0.2431 |
| 2nd dose | 1/24(4.17) | 0 | 2/24(8.33) | 1/18(5.56) | 0.7179 | 0 | 1/24(4.17) | 0 | 0 | 1.0000 |
| **Solicited local adverse reactions** | | | | | | | | | | |
| 1st dose | 0 | 1/24(4.17) | 4/24(16.67) | 4/18(22.22) | 0.0354 | 0 | 0 | 1(4.17) | 0 | 1.0000 |
| 2nd dose | 2/24(8.33) | 1/24(4.35) | 1/24(4.17) | 1/18(5.56) | 1.0000 | 0 | 0 | 0 | 0 | 1.0000 |
| **Unsolicited adverse reactions** | | | | | | | | | | |
| 1st dose | 2/24(8.33) | 2/24(8.33) | 4/24(16.67) | 1/18(5.56) | 0.7252 | 1/24(4.17) | 2/24(8.33) | 2/24(8.33) | 2/18(11.11) | 0.9493 |
| 2nd dose | 3/24(12.50) | 3/24(13.04) | 0 | 0 | 0.0843 | 1/20(5.00) | 1/24(4.17) | 4/24(16.67) | 1/18(5.56) | 0.4954 |

Data are presented as number of adverse events/total participants in this group (%)


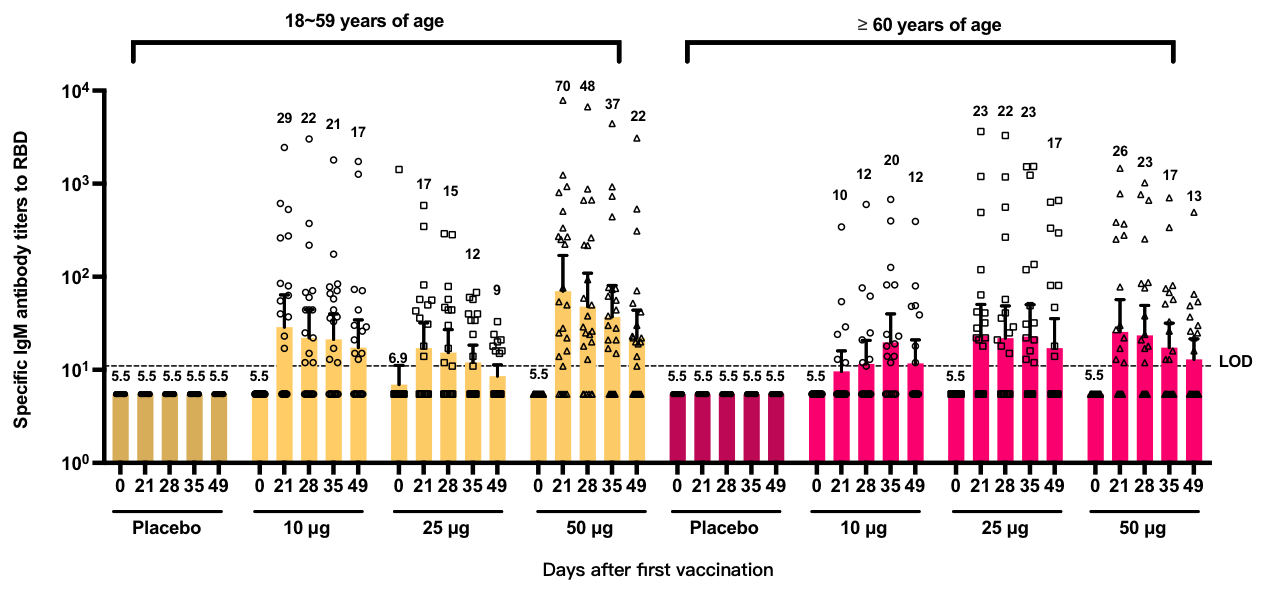


Figure S1: Specific IgM antibody titers to RBD in the phase 1 trial


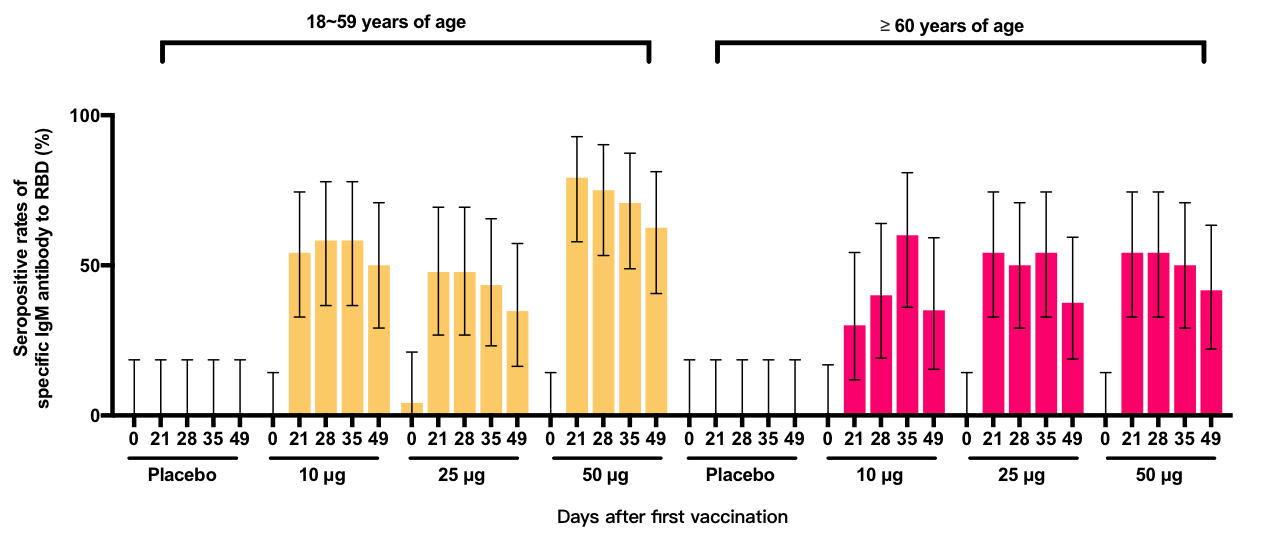


Figure S2: Seropositive rates of specific IgM antibody to RBD in the phase 1 trial

**Supplementary Methods**

**Live SARS-CoV-2 virus amplication and titration**

SARS-CoV-2 virus (BetaCoV/Wuhan/AMMS01/2020 activated, GISAID No. EPI_ISL_5402124）was propagated on Vero E6 cells. The virus was harvested until the cytopathic efficiency (CPE) reached more than 75%. The virus titer was determined also with CPE assay as follows. 1 × 10^4^/well cells were seeded in 96-well culture plate for 18~24 h, and then 10-fold serially diluted virus was added, 6 repeats were set for each dilution, 6 dilutions in all. Cells were cultured in a 5% CO2 incubator at 37°C, and checked under a microscope for the presence of CPE after 4~5 days. Virus titer was calculated with Reed and Muench method.

**Live SARS-CoV-2 neutralization assay**

The cytopathic efficiency (CPE) assay was used to determine 50% neutralization titer to live SARS-CoV-2 . Each serum was incubated at 56 °C for 30 min for inactivation. 1 × 10^4^/well Vero E6 cells were seeded in 96-well culture plate for 18~24 h. On the next day, the inactivated serum was diluted in 3-fold serially started at 1:5, and 6 repeats were set for each dilution. 70 μL serially diluted sera were mixed with 70 μL 140 TCID50 virus per well in 96-well plates, then the sera/virus mixture was incubated at 37°C (5% CO_2_) for 2 hour before transferring 100 μL mixture to 96-well titer plates with confluent Vero E6 cells. After the plates incubating for 4 days, the CPE of each well was recorded under microscopes, and the neutralizing titer was calculated by the dilution number of 50% protective condition using Reed and Muench method.

**Measurement of specific IgG binding to SAR-CoV-2 RBD**

Specific IgG antibody binding to SARS-CoV-2 RBD was measured by indirect ELISA assay using the kits from WANTAI BioPharm. In brief, 96-well plates were coated with purified SARS-CoV-2 RBD protein and blocked in advance. After inactivation at 56 ℃ for 30 min, the sera were subject to serial dilution in 2-fold with a 1:11 dilution as a starting concentration, and then applied to the 96-well plates for 30 min incubation at 37 ℃. After washing, anti-human IgG labeled by HRP was added, followed by sequential addition of substrate solution and stop solution. When the reaction stopped, the absorbance was read at 450 nm and 630 nm. Value of cutoff was 0.16 plus average value of negative control. IgG titer was calculated as the dilution factor of endpoint multiplied value of endpoint and divided by value of cutoff. Samples with values ≥ 11 were defined as seroconverted, while for those positive on day 0, four times more than baseline were defined as seroconverted.

**Measurement of specific IgM binding to SAR-CoV-2 RBD**

Specific IgM antibody binding to SARS-CoV-2 RBD was measured by Magnetism particulate immunochemistry luminescence method (CLIA) using the kits from Antobio. During the detection, the anti-human-IgM-enzyme-labeled antigen complex was formed through immune reaction, which catalyzed the emission of photons from the luminescent substrate. After inactivation at 56℃ for 30 min, the sera were diluted to 1:11 and then detected using Auto Lumo A2000 Plus. While sample/cut off (S/CO) values were higher than 8, further dilution was needed till the S/CO value was between 1 to 8. While S/CO values <1, the result was defined as negative, otherwise, the IgM titer would be calculated as the dilution factor of endpoint multiplied S/CO value. The lower limit of detection was 11, and those below the detection limit were assigned to 5.5.
